# How components of facial width to height ratio differently contribute to the perception of social traits

**DOI:** 10.1371/journal.pone.0172739

**Published:** 2017-02-24

**Authors:** Manuela Costa, Guillaume Lio, Alice Gomez, Angela Sirigu

**Affiliations:** Institute of Cognitive Science Marc Jeannerod, UMR5229, CNRS, UCBL, Lyon 1, Bron, France; Universitatsklinikum Tubingen, GERMANY

## Abstract

Facial width to height ratio (fWHR) is a morphological cue that correlates with sexual dimorphism and social traits. Currently, it is unclear how vertical and horizontal components of fWHR, distinctly capture faces’ social information. Using a new methodology, we orthogonally manipulated the upper facial height and the bizygomatic width to test their selective effect in the formation of impressions. Subjects (n = 90) saw pair of faces and had to select the face expressing better different social traits (trustworthiness, aggressiveness and femininity). We further investigated how sex and fWHR components interact in the formation of these judgements. Across experiments, changes along the vertical component better predicted participants' ratings rather than the horizontal component. Faces with smaller height were perceived as less trustworthy, less feminine and more aggressive. By dissociating fWHR and testing the contribution of its components independently, we obtained a powerful and discriminative measure of how facial morphology guides social judgements.

## Introduction

Facial perception is largely influenced by detection of emotions. However, it is also influenced by morphological and stable factors such as gender, skin color and facial width to height ratio (fWHR). Among these factors, fWHR has recently received great attention [1, 2]

Researches in social psychology showed that fWHR is used implicitly to form social judgments from facial appearance. Male faces with higher fWHR are more likely to be judged as untrustworthy[3, 4], dominant[5, 6], more powerful and competent[7]. Strikingly, this measure can have strong impact on real life since it has been considered in judicial context where the prisoners’ fWHR contributed to the jury’s decision[8]. A link between fWHR and behavioral tendencies has also been established. For example, it has been suggested that men with higher fWHR have a higher propensity to aggression [9, 10], they are more likely to show unethical behavior such as deception, cheating [11] self-interest [12]or little propensity to trust others[4].

Recent studies however have not replicated these findings[13–16]. Deaner et al. (2012) found that body weight, and not fWHR, predicts aggression in hockey players. Along the same line, Gómez-Valdés et al. (2013) did not find any link between fWHR and bellicose tendencies in male mexican prisoners. Moreover, as pointed by Geniole and colleagues (2012) data are lacking concerning the generalizability of this effect for female faces. In line with this, Carré and McCormick (2008) found a relationship between fWHR and aggressiveness in man only. The lack of fWHR effect for woman’ faces has been also reported by Stirrat and Perret (2010) when studying trustworthiness or by Haselhuhn and Wong when investigating unethical behavior (2012).

Such contrasting findings may suggest that fWHR isn’t perhaps a reliable facial dimension strong enough to influence social perception. Others may argue that disparity of results could also reflect differences in the procedure used among the different studies given that no strict consensus exists on how exactly measure fWHR. In other words, we might still lack a validated fWHR method as a standard of face metric.

FWHR is a combined measure obtained by dividing byzigomatic width, the distance between the left and right zygion of the face, by upper facial height, distance between the upper lip and mid-brown. Importantly, no study has yet controlled the effect of each component to investigate how they distinctly contribute to the formation of social impressions

There is evidence supporting a link between upper facial height and some human characteristics (behavior, sex), independently from facial width. Upper facial height, and not facial breadth, is a potential target of selection during evolution [17]. In fact, Weston and colleagues (2007) reported that facial height can unambiguously distinguish an adult male from a female face, whereas the facial width may fluctuate with variation in body size. Despite signaling sex differences, upper facial height may also reveal other characteristics. In fact, faces with smaller upper height have been shown to display more bite force, a trait that may play a crucial role in survival [18, 19]. These studies suggest that variations on byzigomatic width or variations of upper facial height can modify fWHR and produce different percepts.

The objective of this study is to show that fWHR can become a more powerful tool when considering upper facial height (vertical component) and byzigomatic width (horizontal component) as independently contributing to people perception of others’ personality. Hence, in the present study we investigated the independent and combined contribution of vertical and horizontal components of fWHR during the formation of social judgements of trustworthiness, femininity and aggressiveness. In the light of the above findings we expect the vertical component to be a key modulator of femininity, aggressiveness and trust judgments, more than the horizontal component or the fWHR as a global measure. Additionally, given the lack of effect for female faces as previously reported[1, 3, 9, 20], we investigated the role of fWHR for trustworthiness, femininity and aggressiveness judgements using a female database. If gender bias social perception, we expect differences in vertical and horizontal manipulation when using female faces.

## Materials and methods

**Fig 1:**
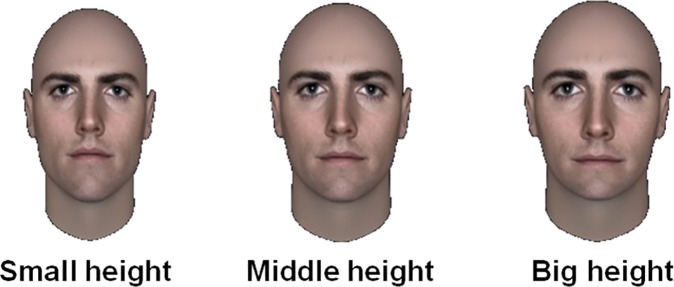
Can faces with the same fWHR trigger different social perception, *i*.*e*. who is more trustworthy, less aggressive or more feminine? Example of three faces selected from the database used in the present study. These faces have the same facial width to height ratio (fWHR) obtained by differently combined upper facial height and bizygomatic width.

To test the effect of each dimension, we orthogonally manipulated the upper facial height (vertical component) and the bizygomatic width (horizontal component) to disentangle their contribution to face impression. Consider for few seconds the three faces in Fig 1, do they represent the same level of trustworthiness or aggressiveness or femininity? These faces have exactly the same fWHR but the size of each component differs. Hence, as Fig 1 suggests face with identical fWHR but with different combination of the two components (vertical and horizontal) can trigger different social judgments.

### Participants

French-speaking participants with normal or corrected vision (n = 90; age between 21 and 43 years, M = 27.6 and SD = 5.8) were recruited for this study. The human study was approved by the local ethical committee Centre Léon Berard, Lyon Sud Est, France, n°ID RCB 2014-A01894-43). Prior to the inclusion in the study, a written informed consent was obtained from all participants. Subjects were randomly selected to attend the trustworthiness, femininity or aggressiveness experiment, using either male or female database (3 social judgments X 2 sex of database used) resulting in 6 independent groups of subjects. In each group we recruited 7 males and 8 females. Since the method employed in the present study is new, we could not refer to previous literature for calculating the effect size. We thus reasoned that a sample size of 30 participants, would achieve 95% power to detect an effect size of f = 0.24 (f<0.25, small effect, conservative choice) with an alpha of 0.05 [21] using G*Power software (v3.9.1.2) [22]

### Material

The original dataset was composed of three male and three female Caucasian faces selected from the Nimstim database[23]. All stimuli were oriented straight and with a neutral expression. Eyes positions were aligned.

**Fig 2:**
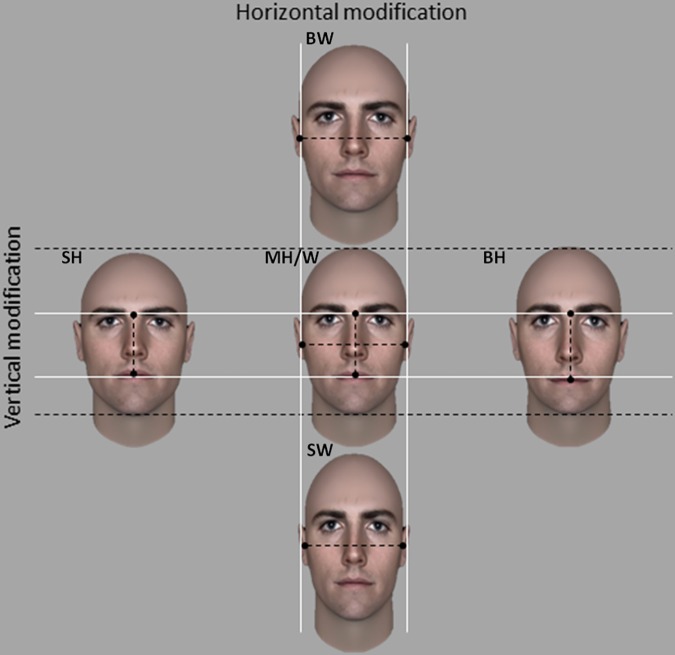
Vertical and Horizontal modification. Examples of computer-generated identities modulated for the upper facial height (vertical modification) and byzigomatic width (horizontal modification). From left to right, vertical modification: Small Height face (SH) corresponds to +3 in FaceGen Modeller (face-brown-chin ratio); Middle Height face (MH), corresponds to 0 zero and Big Height face (BH) corresponds to -3. From top to bottom, horizontal modification: Big Wide (BW), Middle Wide (MW), Small Wide faces (SW).

To generate the vertical modification, the upper facial height of each face was manipulated using the “face-brow-nose-chin-ratio” of FaceGen Modeller 3.5. Three categories of vertical faces were created (Fig 2): 1) the small height faces (SH; 184 px), corresponding to +3 in FaceGen Modeller; 2) the middle height faces (MH; 198 px) corresponding to 0 in FaceGen Modeller; 3) the big height faces (BH; 206 px) corresponding to -3 in FaceGen Modeller.

To generate the horizontal modification, the bizygomatic width of each category of vertical faces (SH, MH, BH) was modulated using Gimp (Version 2.8, http://gimp.org). Three categories of horizontal faces were created (Fig 2): 1) the small wide faces (SW; 324 px); 2) the middle wide faces (MW; 349 px); 3) the big wide faces (BW; 363 px). The degree of horizontal modification was determined using the fWHR (1.76) of the baseline face (MWxMH) and the value of vertical modification previously generated. Thus, big width faces (BW) have a width calculated in a way that the face with the biggest height (BWxBH) has a fWHR of 1.76. Likewise, the small width face has a width (SW) calculated in a way that the face with the smallest height (SWxSH) has a fWHR of 1.76 (S1 Fig). The same methods have been used to modulate female faces.

Following these metrics, each face was modified in 9 different ways, resulting in a final database composed of 54 stimuli (3 vertical x 3 horizontal modifications x 3 identities x 2 conditions, female or male). The same dataset has been used for the six experiments.

Importantly, middle faces have been chosen to represent exactly the median value for faces in the distribution of real population. We then used middle faces as starting point for all modifications; we further assess that also modified faces of our database were still within the same distribution by comparing upper facial height and byzigomatic width of our stimuli with a large sample of real measures. The metric of faces for upper facial height and byzigomatic width for real faces is provided by “FaceBase” database [24]. Z-scores were computed for each category of stimuli generated with respect to FaceBase data (S1 Text, S2 and S3 Figs).

### Procedure

Faces have been presented side by side in a screen of 1920 X 1200 pixels using Matlab, the image resolution was 757 X 820 pixels. Rating of faces consisted in multiple presentations of two randomly selected stimuli from the 27 of the entire Database. All possible couples have been presented for a total number of 351 trials per participant. Participants (n = 15 for each experiment) were requested to select the face expressing better the social trait being tested—trustworthiness, aggressiveness and femininity—by pressing on the keyboard. Participants knew that they were attending a study on first impression where they were encouraged to respond with their “gut feelings”. The score for each face was calculated as the average of all scores obtained from subjects’ choices (face selected = 1; otherwise 0). Because aggressiveness judgments are negatively correlated with trustworthiness and femininity judgements, values reported were then transformed as follow: Used value = 1-observed value to clarify the effect across experiments. This transformation does not modify any of the statistical analysis.

For each experiment, to control variability, the averages across identities have been performed in accord with the vertical component (SH/MH/BH) and the horizontal component (SW/MW/BW). Since sex attributes can bias social judgments (for instance, faces with femininity traits are perceived as more trustworthy than masculine ones [25], we included “type of stimulus” (male/female) as a categorical factor, to assess the effect of stimulus type for each tested category (male or female dataset). Note however that because the primary objective of the study was to test how vertical and horizontal components distinctly capture faces’ social information, we performed a between-group analysis to test this effect.

A mixed ANOVA with vertical and horizontal components as within-subject variables and social traits (trustworthiness-aggressiveness-femininity) and sex of database used (male-female) as between-subjects variables was performed. The ANOVA analysis showed a significant 4-way interaction, F(8, 336) = 2.39; p = 1.59 x 10^−2^. To describe data in a clear manner, we will present results for each effect individually (1: trustworthiness, 2: aggressiveness and 3: femininity). Thus, in each of these mixed ANOVA, the average rating score is assessed with the vertical (SH/MH/BH) and horizontal components (SW/MW/BW) as within subject-variable and the type of stimuli (male or female dataset) as between-subjects.

Effect of linearity has been tested using planned comparison. All post-hoc analyses were done using Bonferroni correction.

## Results

### Trustworthiness judgements

**Fig 3:**
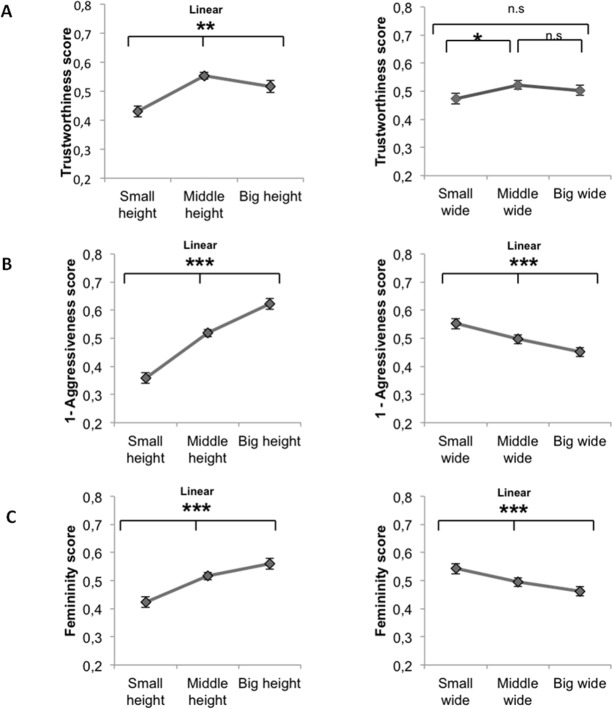
**(A-B-C) Average score for trustworthiness, femininity and aggressiveness judgements in all visual conditions.** Left panels: result for the vertical component (Small-Middle-Big Height faces); right panels: results for the horizontal component (Small-Middle-Big Wide faces). Because aggressiveness judgments are negatively correlated to trustworthiness and femininity judgements, values reported are 1-Aggressiveness values to clarify the effect across experiments. This transformation does not make statistical changes.

A mixed ANOVA showed that perceived trustworthiness of faces was significantly modulated by the vertical component, *F*(2,56) = 13.59, *p =* 1.50 x 10^−5^, η^2^_p_ = .33. Planned comparisons showed a linear effect *F*(1,28) = 7.025, *p =* 1.30 x 10^−2^. Faces with smaller height were judged as less trustworthy (M_SH_ = 0.43, SD = 0.10; M_MH_ = 0.55, SD = 0.06; M_BH_ = 0.51, SD = 0.11) (Fig 3A, left graph). This effect was independent from sex, (male/female database used), as there was no interaction (*F*<1) between sex and the vertical component.

The horizontal component also slightly biased participants perceived trustworthiness of faces, *F*(2,56) = 4.61, *p =* 1.39 x 10^−2^, η^2^_p_ = 0.14. However, planned comparisons did not show a linear effect *F*(1,28) = 1.77, *p* = 1.94 x 10^−1^. Post hoc analysis showed that faces with smaller width were judged as less trustworthy (M_SW_ = 0.47, SD = 0.10; M_MW_ = 0.52, SD = 0.10; M_BW_ = 0.50, SD = 0.11) (Fig 3A, right graph). This effect was independent from sex, (male/female database used), as there was no interaction (*F*<1) between sex and the vertical component.

For trustworthiness judgments, the ANOVA showed a significant interaction between the vertical and the horizontal components: *F*(4,112) = 5.90, *p =* 2.41 x 10^−4^, η^2^_p_ = 0.17. A triple interaction with sex and components (vertical X horizontal X sex), *F*(4,112) = 3.15, *p =* 1.71 x 10^−2^, η^2^_p_ = 0.10 was also observed. Planned comparisons showed that vertical/horizontal interaction was significant for male *F*(4,56) = 7.29, *p =* 8.50 x 10^−5^, η^2^_p_ = 0.34 but not for female faces (*F*<1).

### Aggressiveness judgements

A mixed ANOVA showed that perceived aggressiveness of faces was strongly modulated by the vertical component, *F*(2,56) = 83.1, *p =* 1 x 10^−6^, η^2^_p_ = 0.75. Planned comparison showed a linear effect *F*(1,28) = 90.82, *p =* 1 x 10^−6^. Faces with smaller height were judged as more aggressive (M_SH_ = 0.35, SD = 0.011; M_MH_ = 0.51, SD = 0.08; M_BH_ = 0.62, SD = 0.11) (Fig 3B, left graph). There was a significant interaction between sex and vertical component, *F*(2,56) = 7.70, *p* = 1.11 x 10^−3^, η^2^_p_ = 0.21. Planned comparison showed a significant linear effect for female *F*(1,28) = 21.85, *p =* 6.80 x 10^−5^ and male faces *F*(1,28) = 77.50, *p =* 1 x 10^−6^. The linear difference between the two sex was also significant *F*(1,28) = 8.5, *p* = 6.84 x 10^−3^ *p* < .01.

The horizontal component also biased participants perceived aggressiveness of faces, *F*(2,56) = 18.5, *p =* 1 x 10^−6^, η^2^_p_ = 0.39. Planned comparison showed a linear effect *F*(1,28) = 20.90, *p =* 8.90 x 10^−5^, faces with smaller width were judged as less aggressive (M_SW_ = 0.55, SD = 0.14; M_MW_ = 0.49, SD = 0.14; M_BW_ = 0.45, SD = 0.14) (Fig 3B, right graph). This effect was independent from sex, i.e., database used, as there was no interaction between sex and horizontal component, *F*(2,56) = 2.26, *p =* 1.13 x 10^−1^.

Finally, there was no significant interaction between vertical and horizontal component F(4,112) = 1.33, *p =* 2.60 x 10^−1^, and no triple interaction between components and sex (vertical X Horizontal X sex) *F*(4,112) = 1.41, *p =* 2.34 x 10^−1^.

### Femininity judgements

A mixed ANOVA showed that perceived femininity of faces was strongly modulated by the vertical component, *F*(2,56) = 46.9, *p =* 1 x 10^−6^, η^2^_p_ = 0.62. Planned comparisons showed a linear effect *F*(1,28) = 49.99, *p =* 1 x 10^−6^: faces with smaller height were judged as less feminine (M_SH_ = 0.42, SD = 0.09; M_MH_ = 0.51, SD = 0.06; M_BH_ = 0.56, SD = 0.07) (Fig 3C, left graph). There was a significant interaction between sex and vertical component, *F*(2,56) = 3.39, *p =* 4.09 x 10^−2^, η^2^_p_ = 0.10. Planned comparison, showed, as in the aggressiveness experiment, a linear effect when using both female *F*(1,28) = 13.19, *p =* 1.11 x 10^−3^ and male database *F*(1,28) = 40.54, 1 x 10^−6^. Finally, using the difference between values obtained for the male and female database we observed only a trend for the linear effect *F*(1,28) = 3.75, *p =* 6.31 x 10^−2.^ .

The horizontal component also biased participants perceived femininity of faces, *F*(2,56) = 15.8, *p =* 4 x 10^−6^, η^2^_p_ = 0.36. Planned comparison showed a linear effect *F*(1,28) = 17.97, *p =* 2.21 x 10^−4^. Faces with smaller width were judged as more feminine (M_SW_ = 0.55, SD = 0.08; M_MW_ = 0.49, SD = 0.08; M_BW_ = 0.45, SD = 0.10) (Fig 3C, right graph). This effect was independent from the sex, (male/female database), as there was no interaction (*F*(2,56) = 2.18, *p =* 1.22 x 10^−1^) between horizontal component and sex.

Finally, the interaction between vertical and horizontal components was not significant *F*(4,112) = 2.14, *p =* 7.97 x 10^−2^, but there was a significant triple interaction (vertical X horizontal X sex) *F*(4,112) = 2.47, *p =* 4.88 x 10^−2^, η^2^_p_ = 0.08. Planned comparison showed that the vertical and horizontal interaction was significant for male faces *F*(4,56) = 2.80, *p =* 3.40 x 10^−2^, η^2^_p_ = 0.16, but not for female faces *F*(4,56) = 1.71, *p =* 1.58 x 10^−1^.

In sum, the effect of vertical component in all experiments and conditions was higher compared to the horizontal one and compared to the interaction effects: Trustworthiness experiment (η^2^_p_Vert = .32 > η^2^_p_Inter = .17 > η^2^_p_Hor = .14); Aggressiveness experiment (η^2^_p_Vert = .74 > η^2^_p_Hor = .21; Inter = n.s); Femininity experiment (η^2^_p_Vert = .62 > η^2^_p_Hor = .10; Inter = n.s) (S4 Fig).

#### Inter-subject reliability

**Fig 4:**
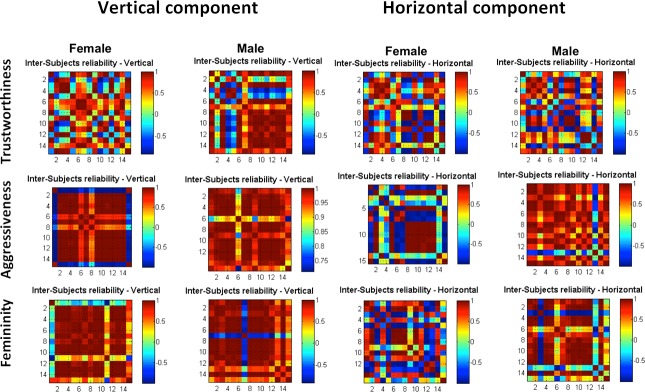
Inter-subject reliability. From left to right, matrixes of correlation for female and male database used for the vertical and the horizontal component. From top to bottom the three social judgments performed during the experiments: trustworthiness, aggressiveness and femininity. Each graph represents a correlation matrix across participants (n = 15 for each experiment). For each participant, ratings of the vertical component (SH, MH, BH) have been correlated to ratings obtained for the three categories of every other participant. The same analysis has been performed using scores of the horizontal component (SW,MW,BW). Colors bar represent the degree of correlation using R from blue (negative correlation, -1) to red (positive correlation, +1). Participants reached higher agreement while judging faces modulated by the vertical component.

To provide further evidence of the relative bias that each component added to social judgments, we assessed the inter-subject-rating reliability. For each participant, ratings of the vertical component (SH, MH, BH) have been correlated with the ratings of the other participants (n = 15) that performed the same experiment. This has been done for each experiment (trustworthiness, aggressiveness or femininity) and for each type of data based used (male/female). Results are presented as a correlation matrix for each experiment (trustworthiness, aggressiveness or femininity) and each database (Fig 4, left graph). The same analysis was conducted using participants’ ratings for the horizontal component (SW, MW, BW), (Fig 4, right graph).

**Fig 5:**
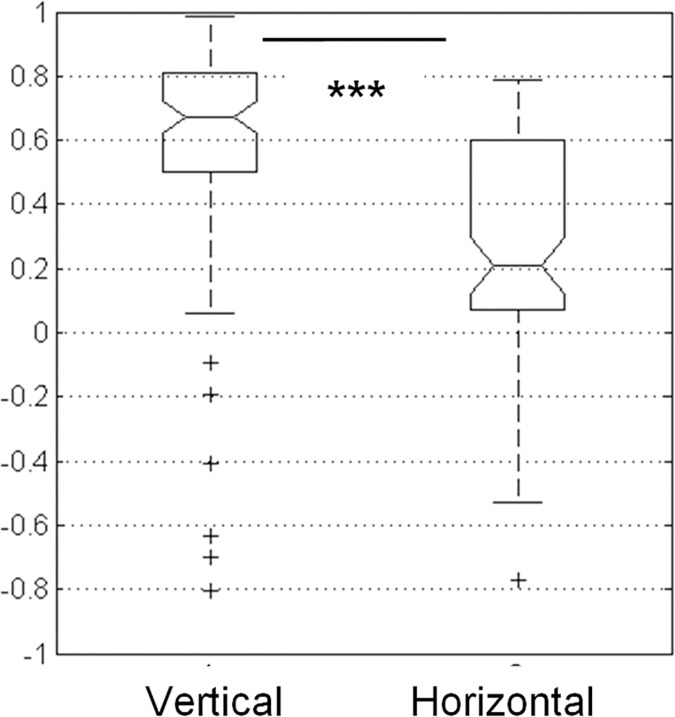
Vertical effect. average of participants’s agreement for the vertical component (modulation of upper facial height) and the horizonthal component (modulation of the bizygomatic width). Sign test showed a strong significant difference *p*< 7.78 x 10^−14^.

We then computed the average of participants’ agreement for the vertical and the horizontal component for each experiment (trustworthiness, aggressiveness, femininity). Because data didn’t follow a normal distribution we performed a non-parametric test (sign test) to compare agreement among participants while judging faces modified for the vertical component (SH-MH-BH) against judgments of faces modified for the horizontal component (SW-MW-BW). The sign test showed a strong effect signaling that the highest agreement was reached for the vertical component *p*<7.78 x 10^−14,^ Fig 5.

## Discussion

Our results show that vertical and horizontal components of fWHR play different roles in the formation of social impression. The methodology employed in this study allowed us to disentangle the facial impression induced by the vertical component from that produced by the horizontal one. We also measured the whole effect of fWHR to assess if the contribution of one component (i.e., vertical) was dependent or independent from the other component (i.e., horizontal).

For judgements of trust, the vertical component strongly affected the attribution of trustworthiness for both male and female faces. Changes in perceived trustworthiness were function of the amount of vertical manipulation: faces with smaller height were perceived as less trustworthy, less feminine and more aggressive compared to faces with bigger height which were perceived as more trustworthy, more feminine and less aggressive. The effect on trust judgement was not affected following horizontal modification while it did when participants judged aggressiveness and femininity. Both the vertical and the horizontal components perceptually affected judgments of femininity and aggressiveness, and this was found for both female and male dataset, although the effect of the vertical component for male faces yielded more significance.

Hence, these results demonstrate that the modulation of the upper facial height is a relevant cue affecting several types of social judgments. In fact, as shown by the size of the eta square, across all experiments and conditions, the vertical effect always better explained participants’ judgments than either the horizontal or the effect of the combined component. This result was also confirmed by the inter-subject reliability analysis, which showed a higher agreement across participants while judging vertically modulated faces.

Why would the vertical component play a more significant role in social judgments?

One explanation may be that upper facial height (and not facial breadth) is a potential target of selection during evolution, as previously argued[17]. In fact, Weston and colleagues (2007) reported that the relationship between bizygomatic width and the usual skull size does not differ between males and females whereas the relationship between upper facial height and skull size significantly differs between the sexes. Therefore, facial height can unambiguously distinguish an adult male from a female faces, whereas the facial width may fluctuate with variation in body size. Therefore, this component may be crucial for judging the face femininity. Despite signaling sex differences, this cue may also reveal other characteristics (S2 Text, S5 Fig). In fact, faces with smaller upper height have been shown to display more bite force which may play a crucial role in survival[18, 19]. As a consequence, it is possible that faces with such characteristics may be perceived and judged as more aggressive. Here, coherently with this literature, we demonstrated that participants strongly relied on the vertical dimension and that faces with small upper facial height have been judged as more aggressive and less feminine but also less trustworthy compared to all other stimuli.

Another possible explanation for the advantage of the vertical component over the horizontal one may be that the upper facial height is less variable than facial width in humans[26, 27]. In fact, facial width but not upper facial height may greatly vary with change in skin quality related to oldness, body size or fat variation. Indeed, it has been already argued that the presence of fat facial tissue in cheekbones makes fWHR difficult to measure[13]. Hence, upper facial height would be a less variable feature and thus easier to perceive from a face than facial width.

In agreement with previous studies, we also found that faces with larger width were judged as more aggressive and less feminine, regardless the sex identity. These results are coherent with previous literature showing that during puberty under the influence of testosterone, males would get larger facial width [17, 28] and that, in return, the faces with larger width would be perceived as more aggressive [29]. Hence, testosterone can be considered as a potential modulator of both physical (width of the face) and behavioural aspects. This step forward was important to clarify the role of biological constraints exert on facial metrics which are relevant for femininity and aggressiveness judgments. Following this reasoning, while taking advantage of our results, future studies may assess the influence of neuromodulators relevant for trustworthiness like oxytocin [30] or those important for aggressiveness such as serotonin [31] on upper facial length.

In all three experiments we did not observe a significant interaction between the vertical and horizontal components when judging female faces on trust, aggressiveness and femininity. This lack of interaction was also observed when judging male faces on aggressiveness. In other words, in most conditions, the effect of the vertical dimension was completely independent from the effect of the horizontal component. Again, this observation is coherent with the hypothesis that selection pressure exerted on facial height is independent from facial width[17].

As a consequence, for future studies, these results strongly support a methodology where the measure of the vertical component *per se* is favored over the complex and less fine-grained measure of fWHR. There are different ways to modulate fWHR, but as it has been shown in this work, it is important to determine which of these two variants enable faces’ first impressions to occur. Based on a previous literature, faces with higher fWHR *tout court* are judged as less trustworthy, more aggressive and less feminine [3, 10]. Our findings rather demonstrate that it is possible to determine which component of fWHR is more relevant in the formation of these social impressions. Future studies may continue to use our methodology to investigate other differences in social traits that are not been considered in the present work. Nonetheless, our findings draw attention on the need to control for these two components when discussing the impact of the fWHR as an integrated measure.

Finally, contrary to previous results that threaten the validity of fWHR as they did not report significant effects using female faces [3, 9, 12, 20] we observed that the vertical effect for trust and the horizontal effect for aggressiveness and femininity were sex-independent.

This study shows that each component of the fWHR is useful to search differences in social perceptions and male-female facial disparities. Altogether our findings suggest that face height (vertical component) and face width (horizontal components) should be tested independently. This method may reduce the ambiguity of using the ratio between these two components.

## Supporting information

S1 Fig Categories of male and female stimuli. Typologies of faces from male (on the left) and female (on the right) dataset for the three visual conditions according to vertical modification (Small Height, Middle Height, Big Height) and to horizontal modification (Big Wide; Middle Wide; Small Wide); color bar represents the FWHR. Big Wide Height, Small Wide Height and Middle Wide Height faces have the same FWHR.
(TIF)

S2 Fig Upper facial height normal distribution in real population. Normal distribution of upper facial height in male population (dark grey) and female population (light grey); from left, sample of Small Height faces (SH), Middle Height faces (MH) and Big Height faces (BH).
(TIF)

S3 Fig Bizygomatic width normal distribution in real population. Normal distribution of bizygomatic width in male population (dark grey) and female population (light grey); from left, sample of Small Wide faces (SW), Middle Wide faces (MW) and Big Wide faces (BW).
(TIF)

S4 Fig Effect size across experiments. The graph reports the effect size using η^2^p for each component—vertical, horizontal and their interaction when it was significant—for each experiment performed: trustworthiness, aggressiveness and femininity.
(TIF)

S5 Fig Male and Female trajectories byzigomatic width (BZW) and of upper facial height (FHT). Values of upper facial height and bizygomatic width provided by FaceBase database. Weston found a significantly difference in the intercept between male and female. The graph shows the absence of this difference using these values.
(TIF)

S1 Text Face stimuli and facial measures in normal population. (PDF)

S2 Text Test of sexual dimorphism using FaceBase data. (PDF)
