# Supplementary figures and images for: How components of facial width to height ratio differently contribute to the perception of social traits
Source: PLoS One. 2017 Feb 24;12(2):e0172739. doi: 10.1371/journal.pone.0172739 (PMC5325523; doi:10.1371/journal.pone.0172739)

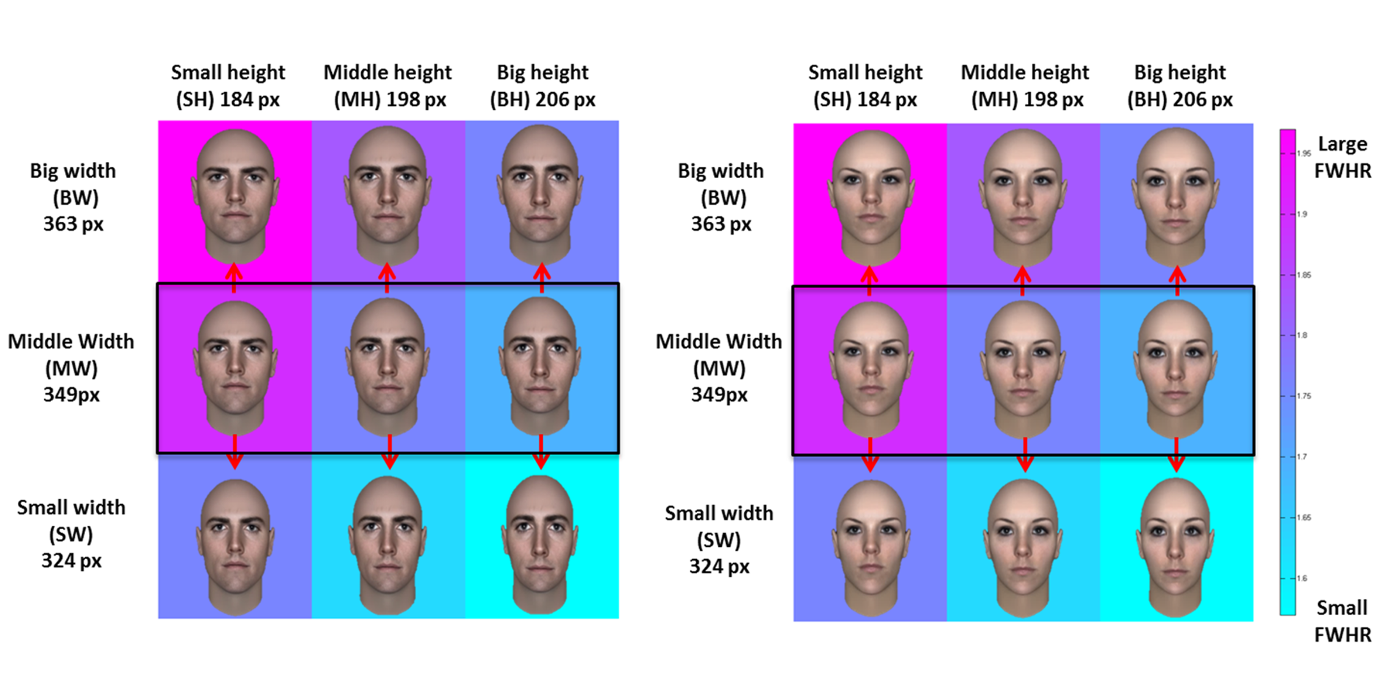

Supplement: S1 Fig — Typologies of faces from male (on the left) and female (on the right) dataset for the three visual conditions according to vertical modification (Small Height, Middle Height, Big Height) and to horizontal modification (Big Wide; Middle Wide; Small Wide); color bar represents the FWHR. Big Wide Height, Small Wide Height and Middle Wide Height faces have the same FWHR. (TIF) [file pone.0172739.s001.tif]

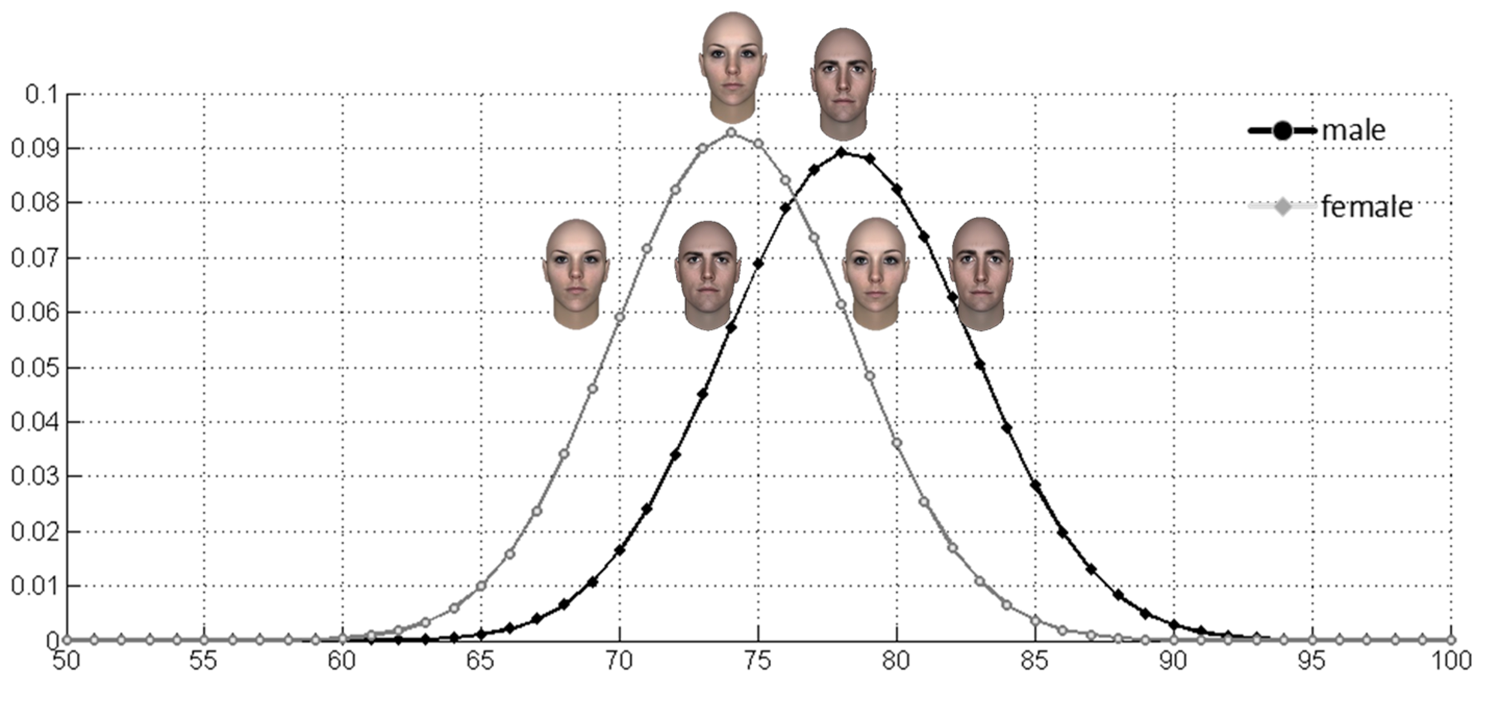

Supplement: S2 Fig — Normal distribution of upper facial height in male population (dark grey) and female population (light grey); from left, sample of Small Height faces (SH), Middle Height faces (MH) and Big Height faces (BH). (TIF) [file pone.0172739.s002.tif]

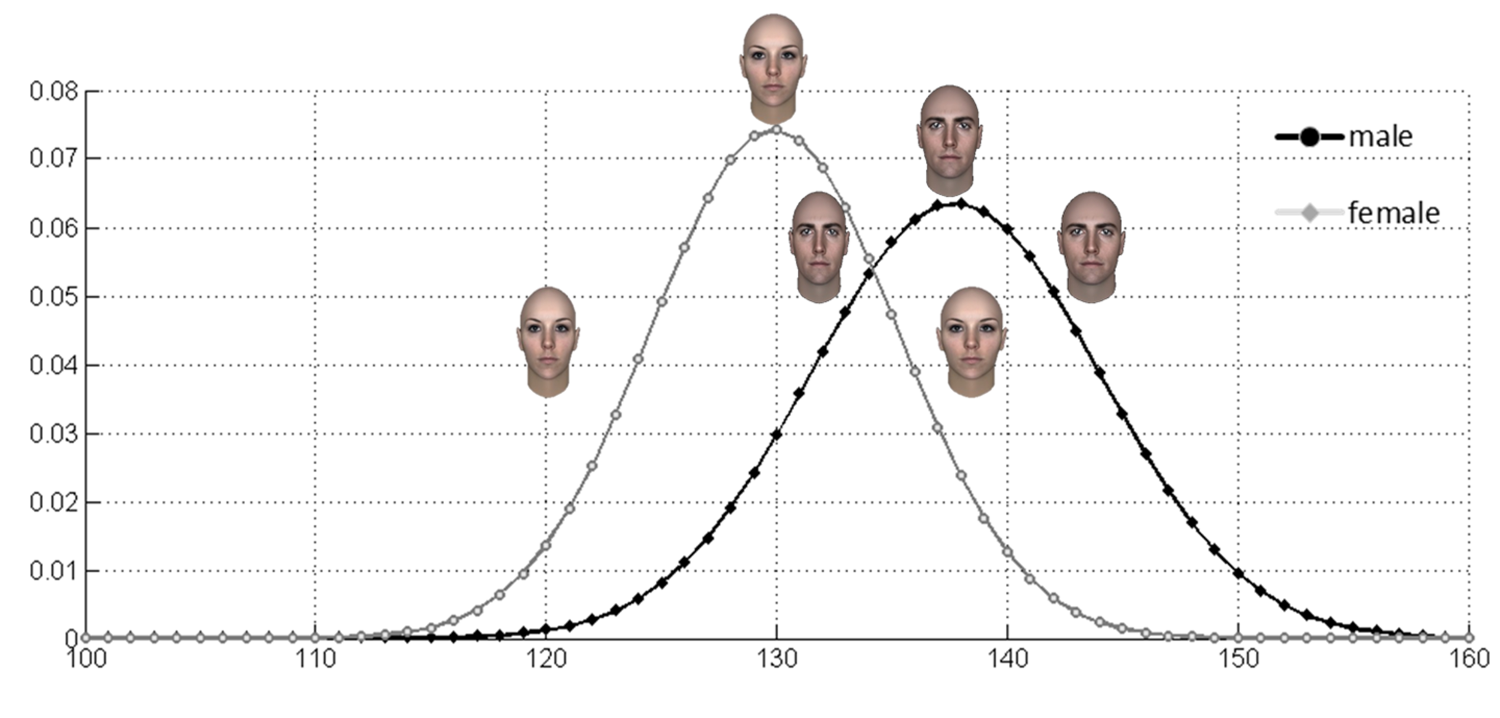

Supplement: S3 Fig — Normal distribution of bizygomatic width in male population (dark grey) and female population (light grey); from left, sample of Small Wide faces (SW), Middle Wide faces (MW) and Big Wide faces (BW). (TIF) [file pone.0172739.s003.tif]

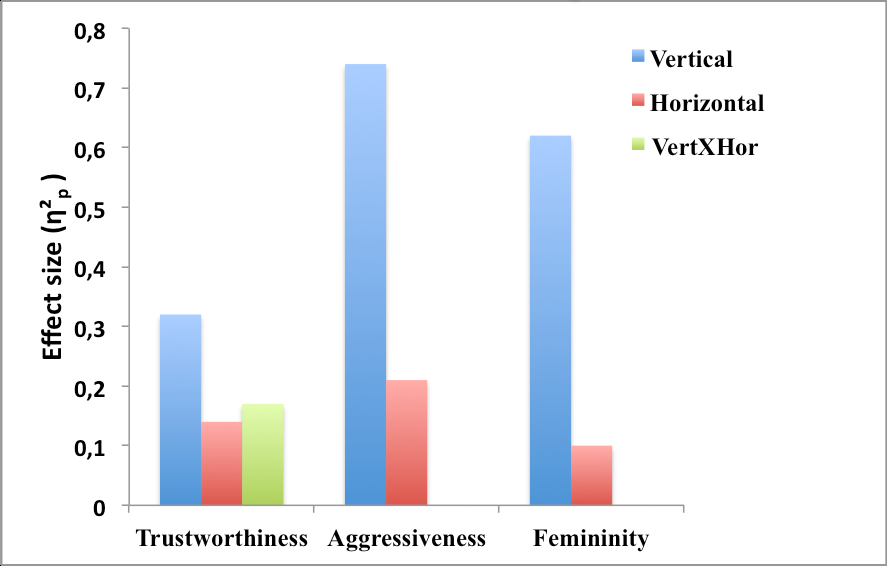

Supplement: S4 Fig — The graph reports the effect size using η2p for each component—vertical, horizontal and their interaction when it was significant—for each experiment performed: trustworthiness, aggressiveness and femininity. (TIF) [file pone.0172739.s004.tif]

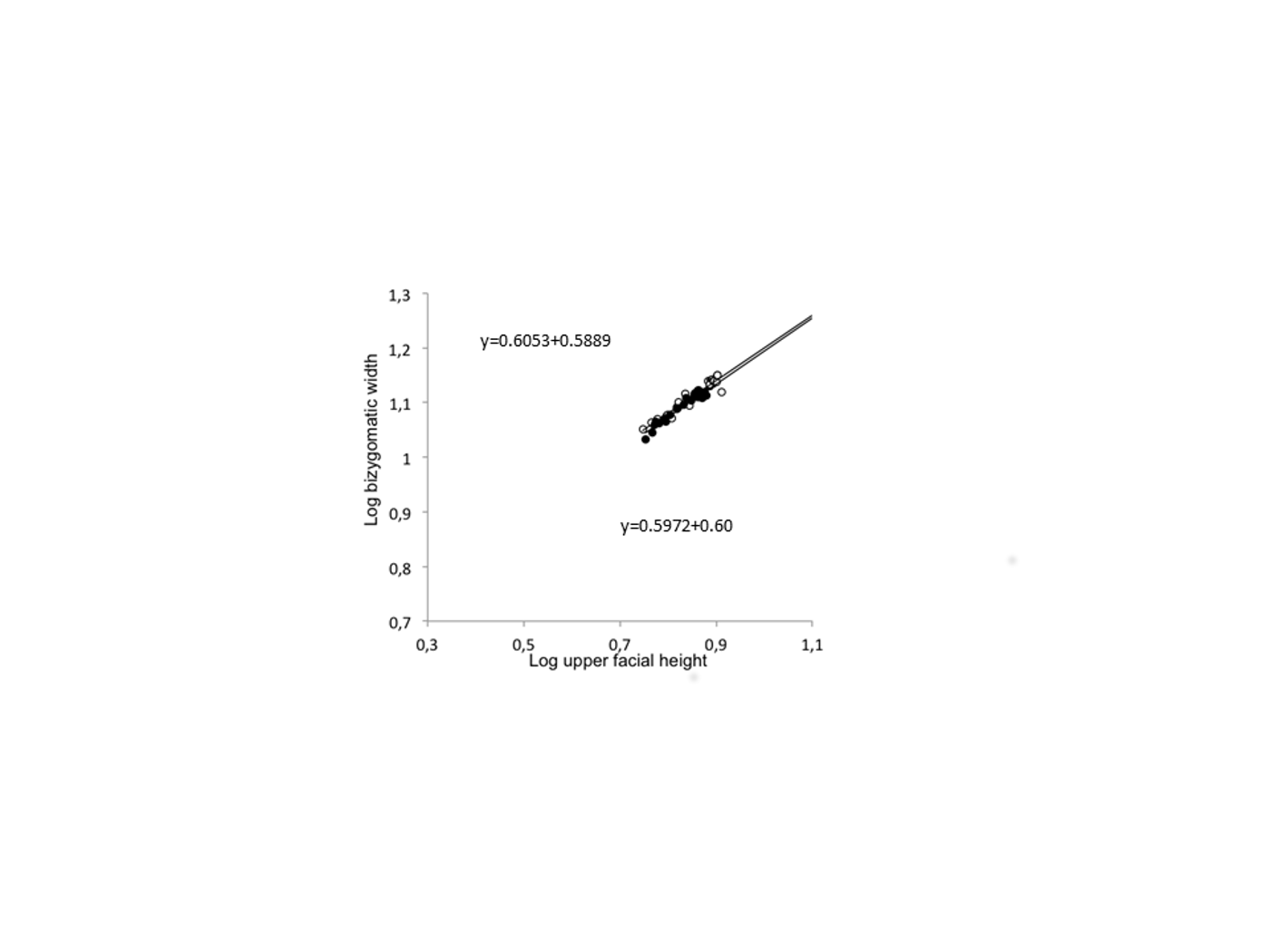

Supplement: S5 Fig — Values of upper facial height and bizygomatic width provided by FaceBase database. Weston found a significantly difference in the intercept between male and female. The graph shows the absence of this difference using these values. (TIF) [file pone.0172739.s005.tif]
