# Supplementary material for: How components of facial width to height ratio differently contribute to the perception of social traits
Source: PLoS One. 2017 Feb 24;12(2):e0172739. doi: 10.1371/journal.pone.0172739 (PMC5325523; doi:10.1371/journal.pone.0172739)
Supplement: S1 Text — (PDF) [file pone.0172739.s006.pdf]

S1 Text. Face stimuli and facial measures in normal population

To insure that stimuli from our dataset were realistic samples from human real faces, our measures of bizygomatic width and upper facial height were compared to the metrics obtained in a normal Caucasian male population provided in the database "FaceBase" (Weinberg et al., 2015). In male, the values selected in Facebase data were the following: (a) upper facial height, *N*=655; M=78.23, SD=4.47; (b) facial width, *N*=614; M=137.76 mm, SD=6.28. The values selected in FaceBase for female: (a) upper facial height, N=1214; M=74.09, SD=4.30; (b) facial width, N=1080; M=129.89, SD=5.37.

We then calculated Z-scores for stimuli in each face category of our database and transformed the value in percentile. We obtained the following results: (a) vertical component, SH=72.7(11%), MH=78.23 (50%), BH=81.42 (76%), (b) horizontal component, SW=128.06 (6%), MW=137.76 (50%), zBW=143.47 (81%) (S2 Fig).

The same analysis has been performed for female faces: (a) vertical component, SH=68.91 (11%), MH=74.15(50%), BH=77.15 (76%); (b) horizontal component, SW=120.5 (4%), MW=129.88 (50%), BW=135.09 (83%). This analysis confirmed that all stimuli used were within the range of the normal population (S3 Fig).
