# Supplementary material for: How components of facial width to height ratio differently contribute to the perception of social traits
Source: PLoS One. 2017 Feb 24;12(2):e0172739. doi: 10.1371/journal.pone.0172739 (PMC5325523; doi:10.1371/journal.pone.0172739)
Supplement: S2 Text — (PDF) [file pone.0172739.s007.pdf]

S2 Text. Test of sexual dimorphism using FaceBase data

Standing from the analysis performed in the previous section we further questioned whether the sexual dimorphism pointed out by Weston (2007) could be found also in our sample and could be replicated using FaceBase database. As argued above Weston and colleagues (2007) reported that the relationship between bizygomatic width and the usual skull size does not differ between males and females, whereas the relationship between upper facial height and skull size significantly differs between the sexes.

However, different studies do not found the sexual dimorphism as reported by Weston (Kramer et al., 2012; Lefevre et al., 2013; Özener, 2012). Coherently with these findings, here we did not find a sign of sexual dimorphism (S5 Fig).

We observed in fact that values of FaceBase for male and female after puberty differed both for their width (Mmale=137.76, SD=6.28; Mfemale=129.89, SD=5.37; $T_{18}=16.21$, $p<.001$), and height (Mmale=78.23, SD=4.47; Mfemale=74.09, SD=4.30; $T_{18}=14.03$, $p<.001$). As a consequence we did not observe a significant difference ($p=0.31$) in the fWHR across sex: Mmale=1.76, SD=0.019; Mfemale=1.75, SD= 0.025. On the contrary difference between variance was significantly different when consider byzigomatic width only ($p<0.5$), upper facial height variance ($p>.05$). A different variance between female and male for the byzygomatic width may be one of the factors that could explain the sexual dimorphism measured by Weston. Another methodological difference was that Weston obtained the measures from skulls.

In line with Ozener (2012) we agree that if fWHR is an important characteristic that emerged as a result of sexual selection in the evolution, evidences for sexual dimorphism should be perceptible from the face (Özener, 2012). In this work, using a new method we found that independently from the existence of sexual dimorphism, modulation of fWHR due to changes in the vertical or in the horizontal component can bias attribution of social judgments from faces both in male and female. We thus strongly suggest further investigating about the existence of sexual dimorphism and the presence of this effect in the female population as two independent but related factors.
